# Early rehospitalization after initial chronic kidney disease educational hospitalization relates with a multidisciplinary medical team

**DOI:** 10.1186/s40780-016-0061-8

**Published:** 2016-10-26

**Authors:** Eiji Kose, Taesong An, Akihiko Kikkawa, Hiroyuki Hayashi

**Affiliations:** 1 Department of Pharmacotherapy, School of Pharmacy, Nihon University, 7-7-1 Narashinodai, Funabashi-shi, Chiba 274-8555 Japan; 2 Department of Pharmacy, Yokosuka Kyousai Hospital, 1-16 Yonegahamadohri, Yokosuka-shi, Kanagawa 238-8588 Japan

**Keywords:** Chronic kidney disease (CKD), Early rehospitalization, Multidisciplinary medical team

## Abstract

**Background:**

It is well-documented that chronic kidney disease (CKD) often results in end-stage renal failure and puts patients at extremely high risk for developing cardiovascular disease. Educational hospitalization at medical institutions in Japan is important for patients with CKD because it facilitates treatment in earlier stages of CKD when subjective symptoms are not apparent. However, some patients who have achieved their educational targets tend to have poor compliance at home after discharge from the hospital, resulting in rehospitalization shortly. In this study, we examined the factors for early rehospitalization of patients after initial CKD educational hospitalization compared with non-rehospitalized patients.

**Methods:**

One hundred thirty-seven patients after discharge from CKD educational hospitalization in Japan between March 2011 and December 2012 were included in the analyses. The subjects were classified into two groups: the early rehospitalization group and control group. We adjusted for confounding variables and performed multiple logistic regression analysis with the presence or absence of early rehospitalization as a dependent variable to investigate the association of early rehospitalization with patient background features, laboratory data, vital signs, instruction-related items, and home environment.

**Results:**

Study subjects included 22 patients in the early hospitalization group and 115 patients in control group. Multivariable analysis for early rehospitalization indicated that insufficient instruction by physician, pharmacist, and dietitians was independent explanatory variable. Analyzing by Kaplan–Meier method, the probability of non-rehospitalization in the instruction group was significantly higher than that in the non-instruction group. Therefore, we believe it is necessary to involve a competent, multidisciplinary medical team (consisting of physicians, pharmacists, and dietitians) in addressing the early rehospitalization issue in patients with CKD.

**Conclusion:**

These findings confirm the importance of care by a multidisciplinary medical team in patients with CKD. Therefore, we suggest that care by a multidisciplinary medical team reduces the increase of early rehospitalization in patients with CKD.

## Background

It is well-documented that chronic kidney disease (CKD) often leads to end-stage renal failure and puts patients at extremely high risk of developing cardiovascular disease (CVD) [1, 2]. Early treatment of CKD reduces the risk of aggravation of renal function, progression to dialysis, and onset of CVD (associated with CKD). However, in a number of patients with early-stage CKD [3] who do not experience subjective symptoms and who do not receive treatment, CKD progresses gradually until renal failure occurs and dialysis becomes necessary. Therefore, detection and early treatment of CKD are imperative in delaying progression of CKD.

To achieve early treatment of CKD, medical institutions in Japan need to provide educational hospitalization to patients with CKD. However, some patients who have achieved their educational targets tend to have poor compliance at home after discharge from the hospital within one year, resulting in rehospitalization after discharge. We examined the factor for rehospitalization within one year after CKD educational hospitalization in previous study [4]. We revealed that rehospitalization within one year after CKD educational hospitalization was not associated with the number of instructions by pharmacists or dieticians but with Alb < 3.5 g/dL, heart failure complications, and eGFR of < 31 mL/min/1.73 m^2^. However, some patients rehospitalized early after discharge were included among rehospitalized patients. We considered that factors other than those revealed in previous study [4] may be involved in these patients. However, in our previous study, we could not analyze the factors for early rehospitalization because the number of patients with early rehospitalization was small. Therefore, in present study, we defined early rehospitalization as rehospitalization within 30 days after discharge from CKD educational hospitalization, and we added the number of patients. Thus, in present study, we examined factors for early rehospitalization of patients after initial CKD educational hospitalization and compared the early rehospitalized patients with non-rehospitalized patients.

### Summary of educational hospitalization

Educational contents of the 2–3-week program were determined in consultations with a team of medical specialists, such as physicians, pharmacists, and dietitians, all of whom had experience in nephrology. During the 2–3-week period, each specialist educated each individual patient on issues pertaining to CKD from their given specialist perspective. We instructed the patients by providing brochures that were prepared by each specialist. Discharge of each patient from the education program was determined by the attending physician after consultations with each of the specialists. Important factors for determining the discharge of each patient included the patient’s level of acquired knowledge from the program and laboratory data on renal function.

## Methods

### Study subjects

We extracted the patients who had been discharged after hospitalization for CKD educational purpose in the Department of Nephrology, Yokosuka Kyosai Hospital from March 2011 to December 2012. The patients discharged within March 2011 to October 2012 were subjected to the analysis in our previous reports [4]. We classified patients who had been rehospitalized within 30 days after discharge from CKD educational hospitalization as early rehospitalization group according to previous report [5]. We defined patients who had not been rehospitalized within 30 days after discharge from CKD educational hospitalization as control group.

### Measurement and collection of clinical data

Data for patients in both the early rehospitalization group and control group were collected at the time of discharge from the educational hospitalization program.

The factors we examined for each patient in both groups were background features (patient characteristics), laboratory data, vital signs, instruction-related items, and home environment. In addition, we classified the patients into Instruction group (instruction by a multidisciplinary team of medical specialists such as physicians, pharmacists, and dietitians) and Non-instruction group (instruction by individual medical specialists such as physicians, pharmacists, and dietitians). We compared the non-rehospitalization rate between both the groups using the Kaplan–Meier method.

### Statistical analysis

Statistical analysis was performed using JMP^®^ (Version 10, SAS Institute Inc., Cary, NC, USA). Our results are presented as mean ± standard deviation (SD). We performed the normality test to compare the data volume between the two groups. We used Student’s *t*-test for data that showed a normal distribution; we used the Mann–Whitney *U*-test for data that did not show a normal distribution. We used the *χ* ^2^ test or Fisher’s exact test to compare categorical data. Next, we adjusted for confounding variables and performed multiple logistic regression analysis with the presence or absence of early rehospitalization as a dependent variable to investigate the association of early rehospitalization with patient background, laboratory data, vital signs, instruction-related items, and home environment. We chose significant factors as independent variables on the basis of univariate analysis results. We chose factors (eGFR, Alb) that were reportedly associated with the progression of CKD [4, 6, 7] and factors whose *p* values were small (i.e., instruction by physicians, pharmacists, and dietitians). Alb and eGFR were not significant in univariate analysis. However, these factors were significantly different between rehospitalization group and non-rehospitalization group in previous study [4]. In addition, decrease in the serum Alb level leads to malnutrition and sthenic inflammatory response and the effects of enhancing the renin–angiotensin system due to the progression of renal dysfunction, blood pressure elevation due to fluid retention, and aggravation of arteriosclerosis are associated with CVD. Thus, we considered that these factors are medically important factors and selected them.

We compulsorily incorporated configuration factors “Subjects who were instructed” into regression equation. We confirmed by multiple logistic regression analysis when no multicollinearity existed between factors using Pearson or Spearman's rank-correlation coefficients. Plots of the estimated probability of non-rehospitalization over time were constructed by the Kaplan–Meier method and were compared with the use of the log rank test. We used the Cox proportional hazards model to calculate hazard ratio. The significance level was *p* < 0.05.

## Results

### Subjects

We extracted 137 patients who had been discharged after hospitalization for CKD educational purposes in the Department of Nephrology, Yokosuka Kyousai Hospital from March 2011 to December 2012. Among the 137 patients, 105 have been subjected to our previous report [4]. Twenty-two patients are classified as early rehospitalization group, and the rest of 115 patients are classified as control group according to previous study [5].

### Patients’ background features and laboratory data

Table 1 shows comparisons of patients’ background features between the early hospitalization group (male, *n* = 10; female, *n* = 12) and control group (male, *n* = 74; female, *n* = 41). No significant differences were noted between the two groups in age, sex, BMI, CKD stage, educational hospitalization period, number of oral drugs, smoking history, and complications (i.e., diabetes mellitus, hypertension, heart failure, CVD, dyslipidemia). BWs for the early rehospitalization group and control group were 50.4 ± 11.0 kg and 58.3 ± 11.0 kg, respectively, with significantly lower values in the early rehospitalization group. Table 2 shows comparison of laboratory data between the two groups. No significant differences were noted in TP, Alb, TLC, CRP, Hb, Hct, HbA1c, BG, LDL-C, HDL-C, TC, TG, K, Ca, P, BUN, Scr, eGFR, UP/Ucr, SUA, SBP, DBP, and PP.

**Table 1:** Comparison of patient background features between early rehospitalization group and control group

Variable	Early rehospitalization group (*n* = 22)	Control group (*n* = 115)	*p* value
Age (y)	73.5 ± 3.0	67.8 ± 1.3	0.0925
Sex (Male/Female) *n*, (%)	10 (45.5)/12 (54.5)	74 (64.4)/41 (35.7)	0.0955
Body weight (kg)	50.4 ± 11.0	58.3 ± 11.0	0.0262
BMI (kg/m^2^)	20.5 ± 3.7	23.1 ± 4.1	0.0914
CKD stage *n*, (%)			
G2	1 (4.6)	2 (1.7)	
G3a	4 (18.2)	22 (19.1)	
G3b	2 (9.1)	26 (22.6)	0.3490
G4	15 (68.2)	59 (51.3)	
G5	0 (0)	6 (5.2)	
Educational hospitalization period (d)	23.2 ± 28.1	20.2 ± 15.8	0.4705
No. of oral drugs	7.8 ± 3.5	8.3 ± 3.7	0.5108
Smoking history *n*, (%)	7 (33.3)	61 (57.0)	0.0660
Complication *n*, (%)			
Diabetes mellitus	8 (36.4)	58 (53.2)	0.1494
Hypertension	12 (54.5)	79 (72.5)	0.0957
Heart failure	6 (27.3)	22 (20.2)	0.4594
Cardiovascular disease	6 (27.3)	42 (38.5)	0.3174
Dyslipidemia	3 (13.6)	38 (34.9)	0.0502

mean ± standard deviation

**Table 2:** Comparison of laboratory data between early rehospitalization group and control group

Variable	Early rehospitalization group (*n* = 22)	Control group (*n* = 115)	*p* value
TP (g/dL)	5.9 ± 0.2	6.1 ± 0.1	0.3166
Alb (g/dL)	3.0 ± 0.7	3.1 ± 0.7	0.6225
TLC (/mm^3^)	1745.1 ± 821.2	1550.7 ± 817.8	0.3214
CRP (mg/dL)	0.9 ± 1.9	0.9 ± 1.9	0.8880
Hb (g/dL)	10.4 ± 2.2	10.8 ± 2.0	0.4828
Hct (%)	32.0 ± 6.6	32.3 ± 6.1	0.8000
HbA1c (%)	5.2 ± 1.5	6.2 ± 1.0	0.1088
BG (mg/dL)	104.1 ± 9.3	121.5 ± 41.3	0.0867
LDL-C (mg/dL)	100.3 ± 27.7	113.6 ± 80.3	0.6646
HDL-C (mg/dL)	40.0 ± 14.4	50.8 ± 20.5	0.4683
TC (mg/dL)	174.2 ± 40.9	203.2 ± 81.6	0.4362
TG (mg/dL)	101.2 ± 49.8	164.1 ± 14.7	0.2294
K (mEq/L)	4.3 ± 0.5	4.1 ± 0.6	0.1259
Ca (mg/dL)	8.5 ± 0.4	8.5 ± 0.7	0.6952
P (mg/dL)	3.5 ± 0.8	3.6 ± 1.0	0.8526
BUN (mg/dL)	34.4 ± 16.7	31.6 ± 17.8	0.5140
Scr (mg/dL)	1.8 ± 0.7	2.1 ± 1.4	0.2347
eGFR (mL/min/1.73m^2^)	30.6 ± 3.4	31.5 ± 15.7	0.8193
UP/Ucr (g/g•Cr)	1.8 ± 2.1	2.9 ± 3.7	0.2388
SUA (mg/dL)	7.4 ± 2.3	7.3 ± 2.2	0.9272
SBP (mmHg)	126.6 ± 17.6	129.0 ± 18.5	0.5747
DBP (mmHg)	68.9 ± 12.6	70.2 ± 12.2	0.6634
PP (mmHg)	57.7 ± 14.0	58.8 ± 16.3	0.7548

mean ± standard deviation

### Reasons for early rehospitalization

**Fig. 1:**
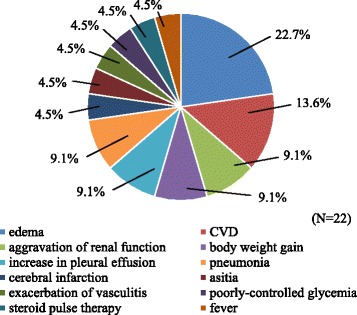
The reason for early rehospitalization in subjects

Figure 1 shows the reasons for early rehospitalization in subjects, with the most common reason being edema, 22.7 %; the second reason was CVD, 13.6 %. Remaining reasons included aggravation of renal function, BW gain, increase in pleural effusion, and pneumonia.

### Comparison of instruction-related items and home environment

Table 3 shows the comparison of instruction-related items and home environment between the early rehospitalization group and control group. The rates of instruction by pharmacists were 40.9 and 64.2 % in the early rehospitalization group and control group, respectively, with a significantly lower rate in early hospitalization group. We also investigated the rate of instruction by physicians, pharmacists, and dietitians to subjects (patients and key specialist) between the two groups. The rates of instruction by physicians, pharmacists, and dietitians were 22.7 and 50.5 % in the early rehospitalization group and control group, respectively. In addition, the rates of subjects who were instructed (patients and key specialist) were 27.3 and 47.3 % in the early rehospitalization group and control group, respectively, with a significantly lower rate in the early rehospitalization group. No significant differences in the number of instruction by pharmacists or dietitians and instruction by physicians or dietitians to subjects (patients only) were observed between the two groups. Furthermore, we investigated spouse or family members in the home environment between the two groups. Rates of spouse presence or absence were 40.9 and 64.2 % in the early rehospitalization group and control group, respectively, with a significantly lower rate in the early rehospitalization group. However, no significant differences in the rate of family members present or absent were observed between the two groups.

**Table 3:** Comparison of instruction-related items and home environment between early rehospitalization group and control group

Variable	Early rehospitalization group (*n* = 22)	Control group (*n* = 115)	*p* value
Instruction by pharmacists *n*, (%)	9 (40.9)	70 (64.2)	0.0415
No. of instruction by pharmacists	1.9 ± 3.3	1.3 ± 1.6	0.2601
Instruction by dietitians *n*, (%)	12 (54.6)	72 (66.1)	0.3046
No. of instruction by dietitians	1.0 ± 1.5	0.7 ± 0.6	0.1015
Instruction by physicians *n*, (%)	21 (95.5)	108 (99.1)	0.2055
Instruction by physicians, pharmacists, and dietitians *n*, (%)	5 (22.7)	55 (50.5)	0.0172
Subjects who were instructed *n*, (%)			
Patients only	16 (72.7)	52 (47.3)	0.0845
Patients and key specialist	6 (27.3)	52 (47.3)	0.0292
Spouse	9 (40.9)	70 (64.2)	0.0415
Family members	15 (68.2)	88 (80.7)	0.1902

mean ± standard deviation

### Multiple logistic regression analysis

**Fig. 2:**
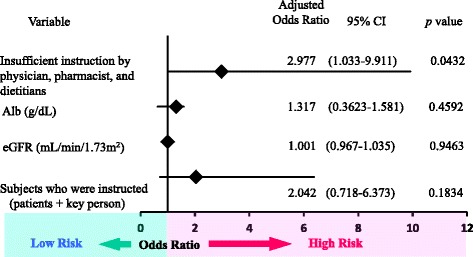
Results of multiple logistic regression analysis of various factors associated with early rehospitalization. 95 % CI: 95 % confidence interval

We evaluated all 137 patients using multiple logistic regression analysis. Various factors associated with early rehospitalization were used in the analysis. Significant differences were observed in the rate of instruction by physicians, pharmacists, and dietitians. Results are listed in a forest plot in Fig. 2.

### Comparison of probability of non-rehospitalization by Kaplan–Meier method

**Fig. 3:**
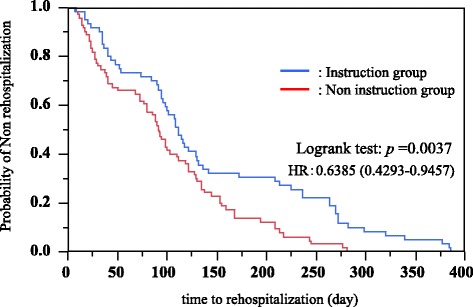
Results of the rate of non-rehospitalization between instruction and non-instruction by physicians, pharmacists, and dietitians using the Kaplan–Meier method. Instruction group: instruction by a multidisciplinary team of medical specialists (i.e., physicians, pharmacists, and dietitians) Non-instruction group: instruction by individual medical specialists (i.e., physicians, pharmacists, and dietitians)

Plots of the estimated proportion of subjects with non-rehospitalization over time were constructed by the Kaplan–Meier method. The log rank test was used to compare the instruction group with the non-instruction group. The probability of non-rehospitalization in the instruction group was significantly higher than that in the non-instruction group. In addition, we calculated the hazard ratio using the Cox proportional hazards model. The hazard ratio of the non-instruction group to instruction group was 0.6385 and showed a low value (Fig. 3).

## Discussion

The most important finding of the present study is the positive association of instruction by physicians, pharmacists, and dietitians with risk of early rehospitalization in patients with CKD. In addition, instruction by a multidisciplinary team of medical specialists significantly extended the period of rehospitalization and suppressed the rehospitalization rate by approximately 36.2 % compared with separate instruction by physicians, pharmacists, or dietitians. Instruction by the multidisciplinary team was more beneficial than instruction by individual medical specialists. These findings confirm the importance of care by a multidisciplinary medical team in patients with CKD. Therefore, we suggest that care by a multidisciplinary medical team reduces the increase of early rehospitalization in patients with CKD.

We examined the factor for rehospitalization within one year after CKD educational hospitalization in previous study [4]. However, some patients who were rehospitalized early after discharge were included among rehospitalized patients. In previous study [4], we could not analyze the factor for early rehospitalization because the number of patients with early rehospitalization was small. Therefore, in the present study, we defined early rehospitalization as rehospitalization within 30 days after discharge from CKD educational hospitalization according to previous study [5] and added the number of patients. We examined factors for early rehospitalization of patients after initial CKD educational hospitalization and compared the early rehospitalized patients with non-rehospitalized patients.

No significant differences were noted in the number of the patients who were instructed by a pharmacist and dietician between the rehospitalization group and non-rehospitalization groups in previous study [4]. This finding is identical to that of the present study. However, in present study, the rates of instruction by physicians, pharmacists, and dietitians in early hospitalization group were significantly lower compared with control group, although the rates of instruction by pharmacists or dietitians were not significantly different between rehospitalization group and non-hospitalization group in previous study [4]. We considered that this difference shows the critical need for aggressive instruction by a multidisciplinary team in early rehospitalization group and suggested that it was necessary to increase the quality rather than the frequency of instructions.

In the present study, the reason why instruction by physicians, pharmacists, and dietitians in the early rehospitalization group was significantly lower value compared with the control group was the large amount of educational content, such as comprehensive instruction on the kidneys, healthy diet, appropriate lifestyle, and adherence to medication and treatment program. We hypothesized that we would not offer this educational content to elderly patients until complete understanding was confirmed, which was one of the factors for analysis. The instruction of patients by the pharmacist and dietitian during educational hospitalization tended to be monotonous and the contents were also often undifferentiated. Therefore, we found it necessary to completely explain the significance of taking medication and correcting lifestyle, and we need to tailor the method of instruction according to individual patients.

The proportion of stage 4 CKD was higher (68.2 %) than that of other CKD stages, and many patients with advanced CKD were included in early rehospitalization group. It has been reported that the risk of end-stage renal failure is higher in patients with advanced CKD, such as stages 4 or 5 CKD [8]. In addition, Keith et al. reported that the rate of patients who required renal replacement therapy was 1.1 % in stage 2, 1.3 % in stage 3, and 19.9 % in stage 4 in their 5-year observational study [9]. Thus, the rate of patients requiring renal replacement therapy was sharply increased when renal function reached stage 4, an obviously important stage in CKD. Actually, the proportion of patients with stage 4 CKD was the highest in the present study. Therefore, we consider that it is necessary to focus on the instruction to the patients with stage 4 CKD. Thus, we investigated the percentage of instruction provided by pharmacists for each CKD stage in the early rehospitalization group and found stage 2 CKD to be 100 %, stage 3a to be 75 %, and stage 4 to be 33.3 %. No value is shown for stage 3b due to the lack of appropriate patients. Stage 4 CKD was the lowest value. Therefore, we also suggested the lack of instruction for patients with stage 4 CKD as a possible cause of early rehospitalization.

There is a report that the rate of side effects increases when the number of oral drugs exceeds six types [10]. Because elderly and CKD patients tend to take more oral drugs, we believe that they are more vulnerable to the development of drug-induced renal failure or drug-induced side effects due to renal dysfunction. Our investigation on the number of oral drugs used in each stage revealed the following: stage 2 = 3.0; stage 3a = 5.3 ± 6.2; stage 3b = 6.0 ± 1.4, and stage 4 = 7.7 ± 2.9, showing that the number of oral drugs increased with progression of CKD stage. In other words, there is a high possibility that renal failure and side effects are expressed in the stage 4 CKD compared with the other stage. However, we could not fully investigate the expression of side effects in this study, and we believed the possibility that this factor potentially involved in the cause of early rehospitalization. Therefore, we recommend that pharmacists should perform more aggressive interventions to delay the start of dialysis or to prevent decline in renal function in the future. Furthermore, we consider it important to identify the subjects who were instructed to maintain adherence to prevent progression of CKD. The subjects who were instructed (patient and key specialist) were not extracted as factors related to early hospitalization in this study. However, in univariate analysis, instructed subjects (patient and key specialist) showed significantly low values in the early rehospitalization group when compared with the control group. In contrast, we found no significant differences between the two groups in the percentage of patient-only instruction (rather than instruction for both patients and key specialists). However, the percentage of instructed patients only was higher in the early rehospitalization group compared with the control group.

The average age of patients with early rehospitalization group in this study was 73.5 ± 3.0 years. Therefore, we noted the difficulty in understanding all educational content when instructing only patients. In contrast, we noted reduced burden and anxiety and better assimilation of knowledge in patients when key specialists were also instructed. We expect that this assimilation of knowledge facilitates better comprehensive care where patients adopt a healthy lifestyle, including diet, exercise, and adherence to medication regimens and follow-up appointments. We realize, however, that as the Japanese population ages (especially elderly patients who live alone), maintaining the aforementioned healthy lifestyle may become more challenging. It is important to remember, therefore, that when we instruct patients, some will find it difficult to adhere to a healthy lifestyle with no support from social resources, a future issue to be considered when identifying instructed subjects.

The present study contains two limitations. (1) It was a single-center cross-sectional study with only a small number of patients undergoing analysis. (2) Although no significant lifestyle-related differences were noted between the two groups, results from the univariate analysis (i.e., history of smoking, hypertension, and dyslipidemia) in the control group tended to be higher than that in the early rehospitalization group. Ninomiya et al. reported that the cumulative incidence of CKD becomes significantly higher with the presence of multiple factors related to metabolic syndrome [11]. Our findings in this respect conflicted with those of Ninomiya et al. One reason could be that of the 115 patients in the control group, 8 had all of these factors, and these factors could have influenced our findings.

## Conclusion

The present study showed that patient instruction by a team of medical specialists (i.e., physicians, pharmacists, and dietitians) was associated with the decreased risk of early rehospitalization in patients with CKD. We realize the challenges physicians face in providing adequate instruction to patients in treatment at various stages of CKD, given the limited consultation time the physicians have in actual clinical practice. To ease these challenges, we recommend patient instruction by a multidisciplinary medical team that includes not only physicians but also pharmacists and dietitians, all of whom are schooled in nephrology and treatment of CKD. Knowing this, we sense a compelling need for the treatment and education of patients with CKD by a multidisciplinary medical team.

## Abbreviations

Alb: Albumin
BG: Blood glucose
BMI: Body mass index
BUN: Blood urea nitrogen
BW: Body weight
Ca: Serum calcium
CKD: Chronic kidney disease
CRP: C-reactive protein
CVD: Cardiovascular disease
DBP: Diastolic blood pressure
eGFR: Estimated glomerular filtration rate
Hb: Hemoglobin
HbA1c: Glycohemoglobin
HDL-C: High-density lipoprotein cholesterol
K: Serum potassium
LDL-C: Low-density lipoprotein cholesterol
P: Serum phosphorus
PP: Pulse pressure
SBP: Systolic blood pressure
Scr: Serum creatinine
SD: Standard deviation
SUA: Serum uric acid
TC: Total cholesterol
TG: Triglycerides
TP: Total protein
Ucr: Urine creatinine
Up: Urine protein
